# *CrystalExplorer*: a program for Hirshfeld surface analysis, visualization and quantitative analysis of molecular crystals

**DOI:** 10.1107/S1600576721002910

**Published:** 2021-04-27

**Authors:** Peter R. Spackman, Michael J. Turner, Joshua J. McKinnon, Stephen K. Wolff, Daniel J. Grimwood, Dylan Jayatilaka, Mark A. Spackman

**Affiliations:** a School of Molecular and Life Sciences, Curtin University, PO Box U1987, Perth, WA 6845, Australia; b School of Molecular Sciences, University of Western Australia, 35 Stirling Highway, Perth, WA 6009, Australia

**Keywords:** intermolecular interactions, Hirshfeld surfaces, fingerprints, visualization, quantum-mechanical properties, computer programs, *CrystalExplorer*

## Abstract

*CrystalExplorer* is a native cross-platform program supported on Windows, MacOS and Linux with the primary function of visualization and investigation of molecular crystal structures, especially through the decorated Hirshfeld surface and its corresponding two-dimensional fingerprint, and through the visualization of void spaces in the crystal via isosurfaces of the promolecule electron density. Over the past decade, significant changes and enhancements have been incorporated into the program, such as the capacity to accurately and quickly calculate and visualize quantitative intermolecular interactions and, perhaps most importantly, the ability to interface with the *Gaussian* and *NWChem* programs to calculate quantum-mechanical properties of molecules. The current version, *CrystalExplorer21*, incorporates these and other changes, and the software can be downloaded and used free of charge for academic research.

## Abstract 2

*CrystalExplorer* is a native cross-platform program for the visualization and investigation of molecular crystal structures.

## 1. Introduction and background   

Rational design of functional materials depends on our capacity to understand the driving forces of binding in the material, along with the mechanism which dictates the desired properties of the material. In the context of molecular crystals, this problem effectively reduces to understanding the strength and origin of intermolecular interactions and their relationship with crystal packing.

Hirshfeld surface (HS) analysis was initiated with the serendipitous discovery of the HS (Spackman & Byrom, 1997 ▸; McKinnon *et al.*, 1998 ▸) as a novel means of partitioning space in molecular crystals into smooth non-overlapping molecular entities. Subsequent developments led to the invention of HS fingerprint plots (Spackman & McKinnon, 2002 ▸; McKinnon *et al.*, 2004 ▸), or ‘fingerprints’ for short. These are a convenient means of summarizing the intermolecular contacts present in crystals via a two-dimensional plot, with the decomposition of this fingerprint plot into features in order to identify particular interactions (McKinnon, Jayatilaka & Spackman, 2007 ▸). The mapping of scalar properties via a colour scale onto the HS (McKinnon, Fabbiani & Spackman, 2007 ▸), known as decoration, has also proven to be a powerful approach to quickly and easily gain insight into molecular environments in the crystalline state.

### 1.1. Prior versions of *CrystalExplorer*   

The *CrystalExplorer* software was initially developed to facilitate HS analysis (Spackman & Jayatilaka, 2009 ▸), and that is still a primary purpose of the program which is currently widely used. However, the ability to perform quantum-chemical calculations, and subsequently generate isosurfaces and surface properties of quantum-mechanically derived quantities (Spackman *et al.*, 2008 ▸), as well as the capacity to identify and visualize void spaces (Turner *et al.*, 2011 ▸) in crystals, has contributed to the popularity of the tool over the past 15 years. These capabilities, made possible through an intuitive graphical user interface available on most desktop computers, have made *CrystalExplorer* into a valuable toolbox for advancing our comprehension and understanding of the structure and properties of molecular crystals.

### 1.2. Changes in *CrystalExplorer21*   

Besides several important bug fixes, the latest version of *CrystalExplorer* now supports use of the open-source *NWChem* (Aprà *et al.*, 2020 ▸) package as a quantum-mechanical back end (through the use of a Molden input file), in addition to the existing support for the *Gaussian* (Frisch *et al.*, 2016 ▸) quantum-chemistry and *Tonto* (Jayatilaka & Grimwood, 2003 ▸) quantum-crystallography packages. Further improvements have been made in finer control over precise views down desired crystal directions, precision in energy tables and more.

## 2. Capabilities   

The aim of this article is to provide a short overview of the capabilities of *CrystalExplorer*; the reader is referred to documentation available online at https://wiki.crystalexplorer.net for further details. *CrystalExplorer* reads a CIF (Hall *et al.*, 1991 ▸) and displays the molecules as ball-and-stick, van der Waals sphere or wireframe models that can be rotated or zoomed into using the mouse wheel.

### 2.1. Crystallographic tools and visualization   

*CrystalExplorer* offers a number of options for building clusters of molecules, allowing the user to see as much or as little of the crystal structure as desired. These include (i) generating molecules close to those on the screen by clicking on nearest-neighbour ‘ghost’ atoms, (ii) generating the remaining atoms of incomplete molecules, (iii) generating all the atoms within a radius of a selection and (iv) generating ‘slabs’ of complete unit cells. The program further includes a selection of tools that are typically found in crystallographic packages, allowing, for example, the calculation of geometric parameters (distances, angles *etc*.), and identification and graphical depiction of short contacts and hydrogen bonds.

### 2.2. Hirshfeld and other surfaces   

HSs, along with what has come to be called HS analysis, have been described in detail in several publications (Spackman & Jayatilaka, 2009 ▸; McKinnon *et al.*, 1998 ▸, 2004 ▸; Spackman, 2013 ▸; Spackman & Byrom, 1997 ▸). They arose from an exploration of possible methods to define a region of the crystal as surrounding a specific molecule, for the purpose of integrating the electron density ‘belonging’ to that molecule.

In *CrystalExplorer*, the electron densities used to compute the HS surfaces come from tabulations of atomic wavefunctions expanded using exponential-type basis functions (Clementi & Roetti, 1974 ▸; Koga *et al.*, 1993 ▸, 2000 ▸). Inspired by Fred Hirshfeld’s ‘stockholder’ partitioning of atoms in mol­ecules (Hirshfeld, 1977 ▸), the resulting (spherical) atomic electron densities 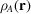 are used to construct the promolecule electron density 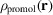 and the procrystal electron density 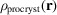, which are then used to define the weight function, 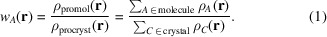
This continuous scalar function has 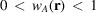 in all space, and the 0.5 isosurface of this function is the HS.

**Figure 1:**
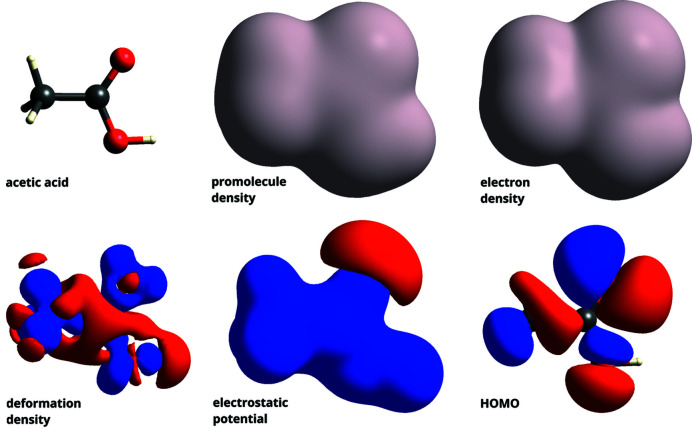
An ensemble of molecular surfaces available in *CrystalExplorer*, displayed using acetic acid (CSD refcode ACETAC01) as an example structure. HOMO refers to highest occupied molecular orbital. All density plots are calculated at the isovalue of 0.002 a.u., while the electrostatic potential surface is calculated at 0.05 a.u. A B3LYP/6-31G(*d*,*p*) wavefunction was used.

Surfaces of interest can be calculated in *CrystalExplorer* by selecting the molecule in question with the mouse and clicking on the HS icon. The user is subsequently prompted to choose the desired surface (and an optional property to decorate the surface) along with a desired surface resolution before the surface is calculated and presented in the three-dimensional graphical display. Fig. 1 ▸ shows the available molecular surfaces using as an example acetic acid [Cambridge Structural Database (CSD) refcode ACETAC01; Nahringbauer, 1970 ▸].

**Figure 2:**
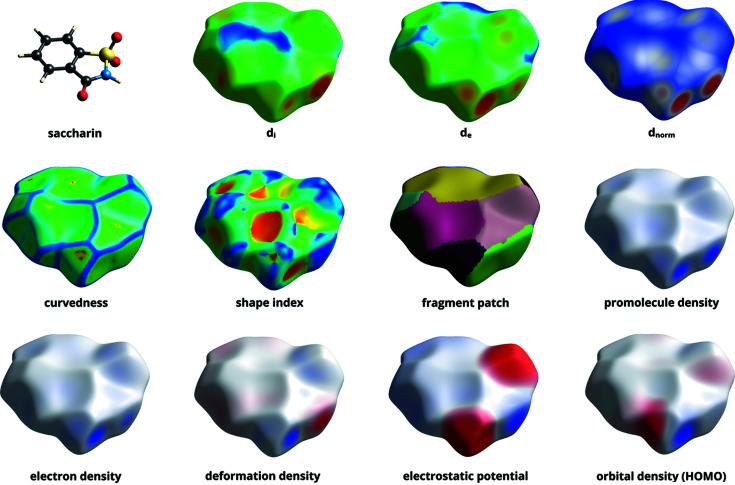
An ensemble of HSs for the saccharin molecule (CSD refcode SCCHRN02; Wardell, Low & Glidewell, 2005 ▸) decorated with different properties available in *CrystalExplorer*. Quantum-mechanical properties were calculated using B3LYP/6-31G(*d*,*p*) wavefunctions.

Molecular surfaces alone provide significant visual information when trying to understand crystal packing, but the addition of scalar ‘surface properties’ mapped to colours decorating the surface further aids in intuitive understanding. Fig. 2 ▸ shows an example of an HS for saccharin, decorated with a variety of properties available in *CrystalExplorer*. The properties themselves are explained in Table 1 ▸. Only the properties on the last row of Fig. 2 ▸ require molecular quantum-mechanical wavefunctions for their evaluation; the other surfaces just make use of geometric distances to the HS, which itself is obtained using only the aforementioned tabulated atomic wavefunctions. Note how qualitatively similar the atom-generated promolecule-density isosurface is to the ‘molecular’ quantum-mechanical electron-density isosurface (Mitchell & Spackman, 2000 ▸).

### 2.3. Fingerprint plots   

*CrystalExplorer* can interactively generate fingerprint plots for all HSs calculated at a sufficiently high resolution. *Crystal­Explorer* ‘links’ the display of the fingerprint to its HS in that a mouse click on the fingerprint plot highlights the corresponding points on the HS (with red cones). Fingerprint plots can be exported in the PNG and PostScript formats, the latter being preferred for inclusion in some publications as it is an editable vector format unlike a rasterized PNG.

*CrystalExplorer* also provides options for decomposing a fingerprint plot into contributions from particular kinds of interactions. At present the user is limited to highlighting interactions on the basis of atom type (C, N, O *etc*.). Interactions are selected by choosing the atom type for the atoms inside the HS and for those outside it. The interactions with the inside and outside atoms switched can also be included by selecting the ‘include reciprocal contacts’ option.

**Figure 3:**
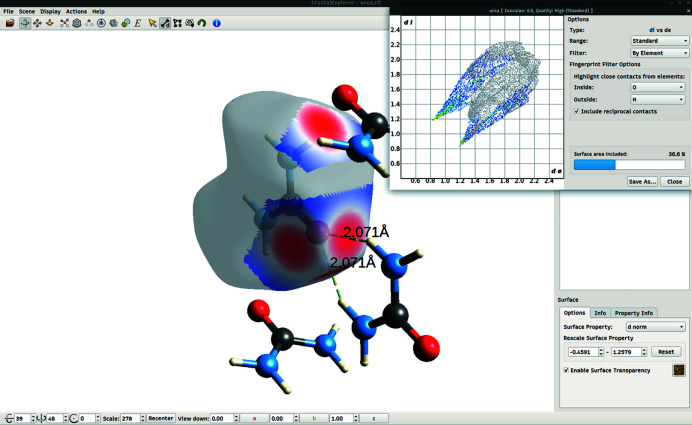
The *CrystalExplorer21* graphical window, showcasing an HS (transparent) surrounding a urea molecule in its crystal environment (CSD refcode UREAXX01), alongside its corresponding fingerprint plot. Neighbouring molecules, corresponding to the highlighted interactions, are also present in the view. O⋯H and H⋯O contacts have been highlighted, demonstrating the link between the regions of the HS and the corresponding values on the two-dimensional fingerprint plot. The coloured fragment patch is decorated with the *d*_norm_ property, and the vectors joining the oxygen atom inside the surface to the two hydrogen atoms outside of it are seen to go through the centre of the red-coloured *d*_norm_ spot.

An example of fingerprint decomposition for urea (CSD refcode UREAXX01; Sklar, Senko & Post, 1961 ▸) can be seen in Fig. 3 ▸. The contacts between oxygen atoms inside the HS and hydrogen atoms (*i.e.* O⋯H contacts) outside of it have been selected, including reciprocal contacts (*i.e.* H⋯O contacts). The parts of the fingerprint that correspond to these interactions remain coloured while the rest of the plot is greyed out, and simultaneously the displayed HS colouring is greyed out with the exception of the regions involving the interaction. The total area of these regions relative to the total surface area of the HS is used to report a percentage contribution of the interaction. We can see from the figure that O⋯H and H⋯O interactions make up 36.6% of the surface area of the HS. There are two fragment patches corresponding to these kinds of interactions and both of them are highlighted by the red-coloured *d*_norm_ property.

### 2.4. *CrystalExplorer* model energies   

*CrystalExplorer* computes pairwise intermolecular interaction energies using an approach inspired by the PIXEL (Gavezzotti, 2005 ▸) method, but the approach used in *Crystal­Explorer* differs in several important points. Full details of the *CrystalExplorer* models are available in the literature (Turner *et al.*, 2014 ▸; Mackenzie *et al.*, 2017 ▸) and only a brief outline is provided here. The basic expression follows that used by Gavezzotti and others, whereby the intermolecular interaction energy is decomposed into four physically motivated terms (explained below) but with the exception that each term is multiplied by its own scale factor: 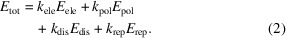
Here *E*_ele_ is the classical electrostatic interaction energy between unperturbed quantum-mechanical charge distributions of the monomers, *E*_pol_ is the polarization energy calculated as a sum over isotropic atomic polarizabilities multiplied by the square of the local electric field caused by the other monomer, *E*_dis_ is Grimme’s D2 dispersion correction (Grimme, 2006 ▸), and *E*_rep_ is the exchange-repulsion energy calculated between unperturbed quantum-mechanical charge distributions of the monomers according to the formula for the sum of the exchange and repulsion terms reported by Su & Li (2009 ▸) and earlier by Hayes & Stone (1984 ▸). The four scale factors in this expression have been optimized for two wavefunction sources: CE-HF (using HF/3-21G monomer wavefunctions) and CE-B3LYP [using B3LYP/6-31G(*d*,*p*) monomer wavefunctions]. These scale factors were optimized against reference intermolecular interaction energies: counterpoise-corrected B3LYP-D2/6-31G(*d*,*p*) interaction energies for 1794 molecule/ion pairs extracted from 171 experimental crystal structures. The resulting models have a mean absolute deviation of 4.7 kJ mol^−1^ for CE-HF and 2.5 kJ mol^−1^ for CE-B3LYP relative to the reference data across an energy range spanning 3.75 MJ mol^−1^ (Mackenzie *et al.*, 2017 ▸).

**Figure 4:**
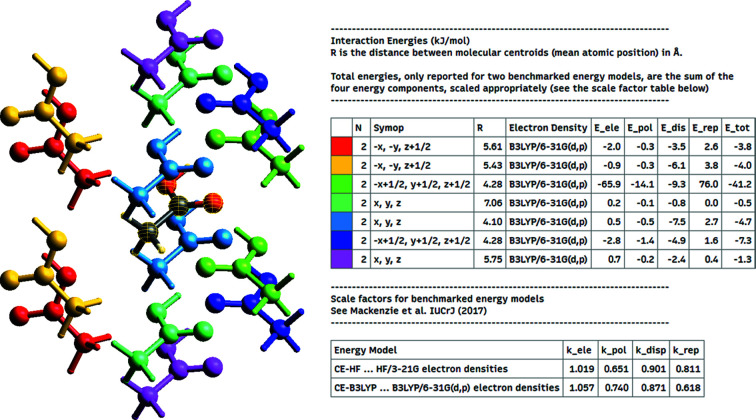
Interaction energies for acetic acid (CSD refcode ACETAC01) calculated with the CE-B3LYP model. It can be seen from the interaction-energies table that the catamer hydrogen-bonding motif between the central molecule (highlighted in yellow mesh) and the −*x* + 1/2, *y* + 1/2, *z* + 1/2 symmetry-related molecule (lime green) is by far the strongest interaction among near neighbours, with an interaction energy of −41.2 kJ mol^−1^.

*CrystalExplorer* uses crystallographic symmetry to determine which interaction pairs are unique. Those that are identical by symmetry are coloured in the same way so that the user may easily see the symmetry-related pairs and their corresponding interactions, along with the crystallographic symmetry operation relating the two molecules. Fig. 4 ▸ shows an example interaction-energy output along with the corresponding graphical view for an acetic acid (CSD refcode ACETAC01) crystal.

**Figure 5:**
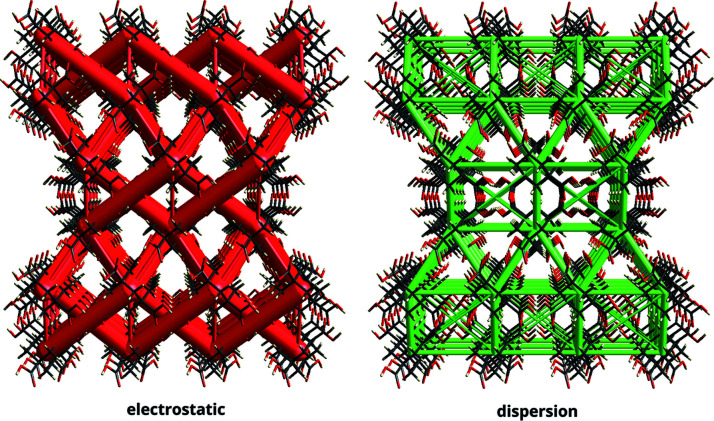
Energy frameworks for a 3 × 3 × 1 supercell, viewed down the crystallographic *b* axis for the fivefold interpenetrated adamantane tetracarboxylic acid (CSD refcode GEJVEW) crystal structure. Despite being a hydrogen-bonded organic framework structure, as demonstrated through the electrostatic framework, we can see a tight cohesive framework of dispersion interactions which also underpin the structure of the crystal.

The interaction energies described above provide quantitative insight into the binding (and non-binding) pairwise intermolecular interactions in the crystal but can be difficult to translate into the context of the actual crystal structure. *CrystalExplorer* alleviates this problem by providing a visualization technique to simplify the information, which we call ‘energy frameworks’ (Turner *et al.*, 2015 ▸). Energy frameworks permit us to examine ‘cooperative effects’ in these intermolecular interactions, across the crystal. Specifically, energy frameworks allowing separate views for the electrostatic (red), dispersion (green) and total energy (blue for binding, gold for non-binding) terms between pairs are available. Fig. 5 ▸ shows an energy-framework visualization for adamantane tetracarboxylic acid (CSD refcode GEJVEW; Ermer, 1988 ▸), a molecular crystal comprising interpenetrated networks of hydrogen bonds.

Furthermore, these intermolecular interaction energies may be directly summed over a number of pairs to give a reasonable estimate of the lattice energy of a crystal (Thomas *et al.*, 2018 ▸; Spackman, 2018 ▸); the CE-B3LYP model energies in particular yield results comparable in accuracy to dispersion-corrected periodic density-functional-theory calculations.

## 3. Availability and installation   

*CrystalExplorer* installers can be downloaded at https://crystalexplorer.net and used free of charge for academic purposes. Commercial licences are also available. *Crystal­Explorer* is supported on Windows 10, MacOS and several GNU/Linux variants. 

## Figures and Tables

**Table 1:** Available surface properties in *CrystalExplorer* r_{I}^{\rm {vdw}} is the van der Waals radius of the nearest atom *I* closest to and inside the HS, while r_{E}^{\rm {vdw}} is the van der Waals radius of the nearest atom *E* closest to and outside the HS. Curvedness and shape index were defined by Koenderink (1990 ▸) and Koenderink & Van Doorn (1992 ▸).

Property	Description
*d*_i_	Distance from HS to the nearest atom *I* internal to the surface
*d*_e_	Distance from HS to the nearest atom *E* external to the surface
*d*_norm_	Normalized sum of *d*_e_ and *d*_i_, *i.e.* (d_{\rm {i}}-r_{I}^{\rm {vdw}})/r_{I}^{\rm {vdw}}+(d_{\rm {e}}-r_{ {E}}^{\rm {vdw}})/r_{E}^{\rm {vdw}}
ρ_promol_	Promolecule electron density
*V*_elec_	Electrostatic potential
ρ	Electron density
|ϕ|^2^	Probability density of a given molecular orbital
ρ_def_	Deformation density, *i.e.* ρ − ρ_promol_
Fragment patch	Unique (coloured) region based on atoms external to the HS designed to indicate the nearest-neighbouring molecule
Curvedness	Function depending on the HS concavity or convexity
Shape index	Function depending on the HS flatness or curvature
